# Supplementary material for: The Role of Ergosterol and Sphingolipids in the Localization and Activity of Candida albicans’ Multidrug Transporter Cdr1p and Plasma Membrane ATPase Pma1p
Source: Int J Mol Sci. 2022 Sep 1;23(17):9975. doi: 10.3390/ijms23179975 (PMC9456455; doi:10.3390/ijms23179975)
Supplement: Supplementary file 1 [file ijms-23-09975-s001.zip › ijms-1862082-supplementary.pdf]

Supplementary Material

The Role of Ergosterol and Sphingolipids in the Localization and Activity of *Candida albicans'* Multidrug Transporter Cdr1p and Plasma Membrane ATPase Pma1p

Aneta K. Urbanek [1,†], Jakub Muraszko [1,†], Daria Derkacz [1], Marcin Łukaszewicz [1], Przemysław Bernat [2] and Anna Krasowska [1,*]

[1] Faculty of Biotechnology, University of Wroclaw, 50-383 Wroclaw, Poland

[2] Department of Industrial Microbiology and Biotechnology, Faculty of Biology and Environmental Protection, University of Lodz, Banacha 12/16, 90-237 Lodz, Poland

* Correspondence: anna.krasowska@uwr.edu.pl

† These authors contributed equally to this work.

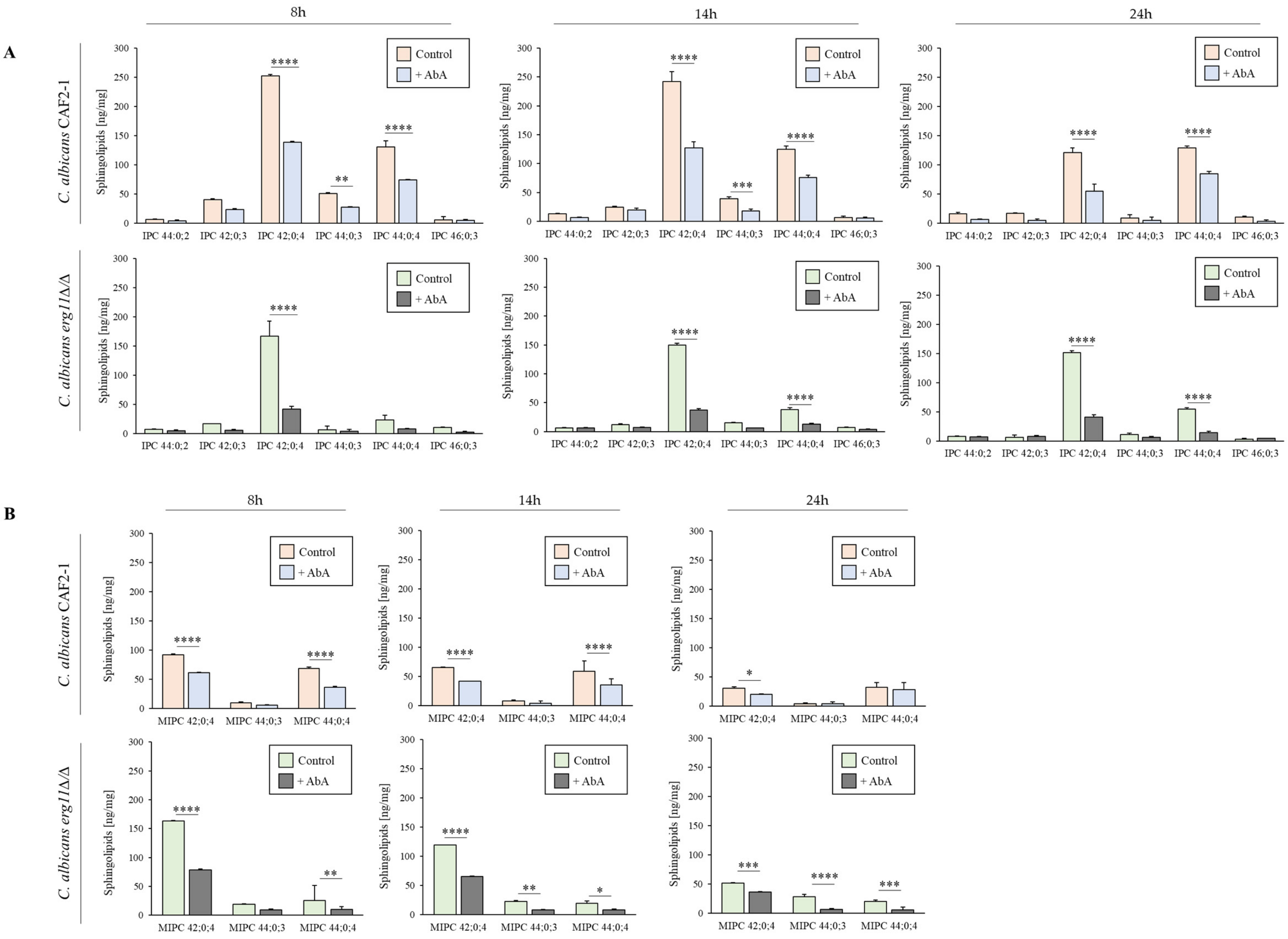

Figure S1. IPC and MIPC species detected in *C. albicans* strains in different growth times (8, 14 and 24h) with AbA (0.005 μg/mL), or without antibiotic (Control, YPD alone). These species are represented as "total number of carbons in the sphingoid backbone and the fatty acyls: total number of double bonds; total number of hydroxyl groups in the sphingoid backbone and the fatty acyls (*, *p<0.05*; **, *p<0.01*; ***, *p<0.001; ****, p<0.0001*).

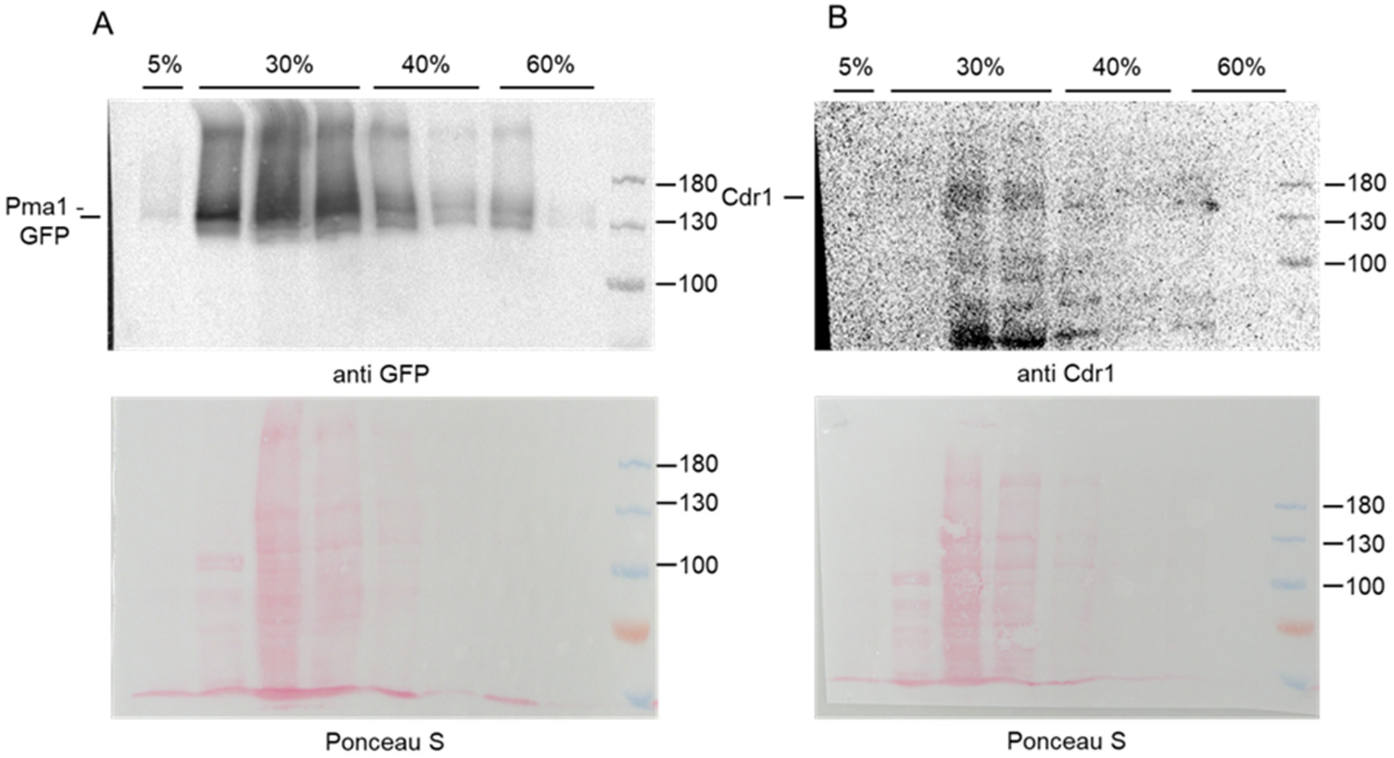

Figure S2. Preliminary test with example of protein content in fractions collected from DRM isolation procedure with Optipreptm gradient (*erg11Δ/Δ* strain, 24h of culturing).
